# Diagnostic accuracy of circulating-free DNA for the determination of *MYCN* amplification status in advanced-stage neuroblastoma: a systematic review and meta-analysis

**DOI:** 10.1038/s41416-020-0740-y

**Published:** 2020-02-04

**Authors:** Ricky M. Trigg, Suzanne D. Turner, Jacqueline A. Shaw, Leila Jahangiri

**Affiliations:** 1 Division of Cellular and Molecular Pathology, Department of Pathology, University of Cambridge, Cambridge, UK; 2 Leicester Cancer Research Centre, University of Leicester, Leicester, UK; 3 Department of Life Sciences, Birmingham City University, Birmingham, UK; 4 Present Address: Functional Genomics, Medicinal Science & Technology, GlaxoSmithKline, Stevenage, UK

**Keywords:** Paediatric cancer, Diagnostic markers

## Abstract

**Background:**

*MYCN* amplification (MNA) is the strongest indicator of poor prognosis in neuroblastoma (NB). This meta-analysis aims to determine the diagnostic accuracy of MNA analysis in circulating-free DNA (cfDNA) from advanced-stage NB patients.

**Methods:**

A systematic review of electronic databases was conducted to identify studies exploring the detection of MNA in plasma/serum cfDNA from NB patients at diagnosis using PCR methodology. Pooled estimates for sensitivity, specificity and diagnostic odds ratio (DOR) were calculated by conducting a bivariate/HSROC random-effects meta-analysis.

**Results:**

Seven studies, with a total of 529 advanced-stage patients, were eligible. The pooled sensitivity of cfDNA-based MNA analysis was 0.908 (95% CI, 0.818–0.956), the pooled specificity was 0.976 (0.940–0.991) and the DOR was 410.0 (−103.6 to 923.7). Sub-grouped by INSS stage, the sensitivity for stage 3 and 4 patients was 0.832 (0.677–0.921) and 0.930 (0.834–0.972), respectively. The specificity was 0.999 (0.109–1.000) and 0.974 (0.937–0.990), respectively, and the DOR was 7855.2 (−66267.0 to 81977.4) and 508.7 (−85.8 to 1103.2), respectively.

**Conclusions:**

MNA analysis in cfDNA using PCR methodology represents a non-invasive approach to rapidly and accurately determine MNA status in patients with advanced-stage NB. Standardised methodology must be developed before this diagnostic test can enter the clinic.

## Background

*MYCN* amplification (MNA) is detected in around 20% of neuroblastoma (NB) patients.^1^ MNA is associated with advanced tumour stage and rapid disease progression, and it is the strongest indicator of poor prognosis for NB.^2^ Methods currently used to determine MNA status include interphase fluorescence in situ hybridisation (FISH), polymerase chain reaction (PCR), multiplex ligation-dependent probe amplification (MLPA) and array comparative genomic hybridisation (aCGH) on tumour material obtained via biopsy.^3^ While FISH has been the gold standard technique for analysis of gene dosage in cancer specimens over the past few decades, it involves subjective evaluation of images by experienced diagnosticians and requires a fluorescent microscope to assess large cell populations.^4^

The biopsy process required for tissue analysis is invasive, and tumours are not always accessible for genetic analysis. Moreover, analysis of biopsy material can be confounded in tumours with an abundance of non-malignant cells^5^ and with heterogeneous patterns of MNA;^6,7^ in recent studies, intratumoural heterogeneity with respect to MNA has been estimated to occur at a frequency of 9.7–10.3%.^8,9^ An alternative approach to tissue-based  MNA analysis involves PCR-based analysis of *MYCN* copy number in circulating-free DNA (cfDNA) isolated from plasma or serum.^10^ This “liquid biopsy” is minimally invasive and may overcome genetic heterogeneity as the method surveys aggregate tumour DNA shed into blood.^11^ In addition, the rapidity of blood processing and PCR analysis enables fast determination of MNA status and assignment of the appropriate therapy for critically ill patients, with a potential sample-to-result turnaround time of less than a day.^12^

The detection of MNA in cfDNA of NB patients was first demonstrated by Combaret et al. in 2002 using a simple qPCR assay targeting *MYCN* and a reference gene (*RPPH1*).^13^ The authors reported high concordance of the MNA status between tumour and serum samples across all disease stages. Subsequent studies have used (q)PCR assays targeting *MYCN* and *NAGK* (also on chromosome 2p) and have consistently reported high sensitivity and specificity for MNA analysis in cfDNA of patients with advanced disease.^14–19^ For example, Yagyu et al. recently reported a sensitivity and specificity of 0.87 (95% CI, 0.72–0.96) and 0.97 (95% CI, 0.84–1.0) among 71 patients with stage 4 NB.^17^ While no clinical trials of NB have formally incorporated cfDNA-based MNA analysis, the aforementioned studies have recruited several hundred patients across multiple disease stages and used similar PCR methodology to measure *MYCN* copy number.^14–19^ Here, we perform a meta-analysis to determine the diagnostic accuracy of MNA analysis in cfDNA from patients with advanced-stage (INSS stages 3 and 4) NB.

## Methods

This meta-analysis was designed and executed in accordance with PRISMA-DTA reporting guidelines.^20^

### Literature search

A comprehensive literature search was undertaken to identify all published studies reporting the sensitivity and specificity of cfDNA-based *MYCN* analysis using PCR methodology. The following electronic databases were searched from inception to August 2019: the Cochrane Central Register of Controlled Trials (CENTRAL), EMBASE, PubMed/MEDLINE and Web of Science Conference Proceedings Citation Index-Science (CPCI-S). The search strategy comprised the terms “neuroblastoma”, “MYCN”, “circulating-free DNA” and terms synonymous with “circulating-free DNA”, including “ccfDNA”, “cfDNA”, “ctDNA”, “cell-free DNA”, “cell free DNA”, “circulating DNA”, “circulating free DNA”, “circulating tumour DNA”, “free DNA”, “free tumour DNA”, “plasma” and “serum”. Keywords were combined using Boolean operators, translated into database-specific syntax, and searched for in the title and abstract only. The search was limited to the English language. Supplementary Information 1 details the search strings used for each database. Additional studies were identified through a manual search of bibliographies in included studies and relevant narrative reviews. Authors of the following publications were contacted by email for further information: Combaret et al.^12,15^ and Yagyu et al.^17^

### Selection criteria

Studies investigating the detection of MNA in plasma or serum cfDNA of NB patients at diagnosis using PCR methodology proceeded to full-text review. The criteria for inclusion were as follows: (1) diagnosis of neuroblastoma confirmed by tumour histology; and (2) matched cfDNA and tumour biopsy material; and (3) use of PCR methodology to detect MNA. The criteria for exclusion were as follows: (1) insufficient data available to determine diagnostic accuracy using 2 × 2 tables (after author contact); (2) absence of disease stage data; and (3) duplicate publication. All included and excluded studies were verified for eligibility by two independent reviewers (R.M.T. and L.J.).

### Data extraction

The following data were independently extracted into an electronic table and assessed by R.M.T. and L.J.: first author name, journal, year of publication, number of patients, baseline patient characteristics (age, gender and INSS tumour stage), blood specimen type (plasma or serum), cfDNA isolation method, *MYCN* PCR method, true positive (TP), false negative (FN), true negative (TN) and false positive (FP) rates.

### Quality assessment

The overall quality of the included studies was determined by two independent reviewers (R.M.T. and L.J.) using QUADAS-2,^21^ a tool developed for the quality assessment of diagnostic accuracy studies. This tool comprises four domains: patient selection, index test, reference standard, and flow and timing, and each domain is assessed for risk of bias and applicability.

### Statistical analysis

MNA status in biopsy tissue as determined by FISH or Southern blot was considered the reference standard. For each study and each INSS tumour stage, 2 × 2 contingency tables were populated with TP (MNA detected in both cfDNA and tumour tissue), FN (MNA detected in tumour tissue but not cfDNA), TN (MNA detected in neither cfDNA nor tumour tissue), and FP (MNA detected in cfDNA but not tumour tissue) data. Diagnostic odds ratio (DOR), sensitivity, specificity, positive likelihood ratio (PLR) and negative likelihood ratio (NLR) were calculated along with corresponding 95% confidence intervals (95% CI) for each study in Meta-DiSc v1.4 statistical software.^22^ Haldane–Anscombe correction^23,24^ was used to avoid errors when dividing by zero in contingency table data, where appropriate. In all, 2 × 2 contingency data were imported into MetaDTA^25^ (https://crsu.shinyapps.io/dta_ma_1_43/), a web-based application for fitting the binomial model of Chu and Cole.^26^ In MetaDTA, the model is fitted as a generalised linear mixed-effect model using the glmer function from the R package lme4.^27^ Percentage study weights were calculated in MetaDTA based on a decomposition of Fisher’s information matrix, according to the recent methodology of Burke et al.^28^ Deeks’ funnel plots were generated by plotting, for each study, the natural logarithm of the DOR against the inverse root of the effective sample size (ESS).^29^ The ESS is calculated from the number of diseased (*n*_*d*_) and healthy (*n*_*h*_) subjects: (4 × *n*_*d*_ × *n*_*h*_)/(*n*_*d*_ + *n*_*h*_). Deeks’ asymmetry test was conducted by linear regression analysis.

## Results

### Studies assessed

**Fig. 1:**
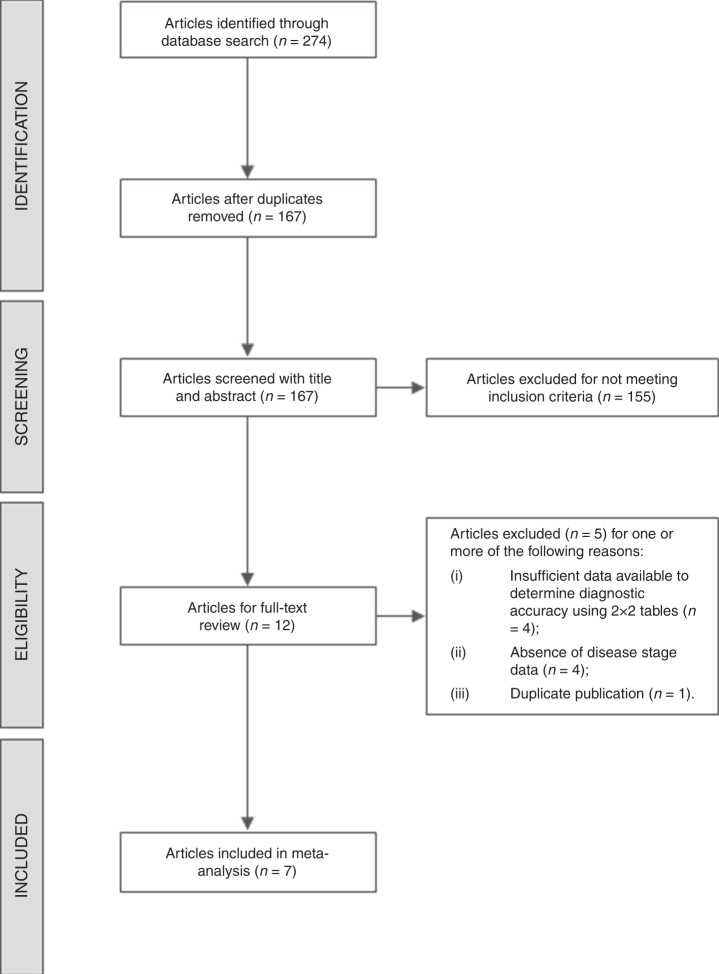
Flow chart for study selection based on PRISMA-DTA guidelines.

A comprehensive search of electronic databases identified a total of 167 studies, with twelve studies reaching the initial criteria for inclusion. Studies were subsequently excluded due to the absence of data required to determine diagnostic accuracy (*n* = 4), absence of INSS stage data (*n* = 4), and duplicate publication (*n* = 1), leaving a total of seven studies for meta-analysis (Fig. 1). These studies, published between 2002 and 2016, recruited a total of 844 NB patients, most of whom were assessed for MNA status at diagnosis by FISH and/or Southern blot of biopsy tissue. All of the studies included employed qPCR (*n* = 6) and/or conventional PCR (*n* = 2) to analyse MNA in cfDNA isolated from plasma (*n* = 2) or serum (*n* = 6) using the QIAamp DNA Blood Kit (Qiagen). In 4/5 studies that reported a cut-off for *MYCN* copy number in cfDNA, a stringent *MYCN*-to-reference ratio of 5.0 could discriminate MNA+ and MNA− patients. The main characteristics of the studies included are summarised in Table 1.

**Table 1:** Main characteristics of the included studies.

Study	Location	Patient *n* by INSS stage (MNA+/MNA−)				Reference standard	Blood specimen	cfDNA isolation kit	MNA cut-off	Assay	Reference gene
		1 + 2	3	4	4S						
Combaret et al.^13^	France	1/24	5/8	25/33	1/5	SB	plasma/serum	QDB kit	NR	qPCR	*RPPH1*
Gotoh et al.^14^	Japan	2/40	2/7	13/18	0/5	SB	serum	QDB kit	(5 to) 10	qPCR	*NAGK*
Combaret et al.^12^	France, Spain	0/25	4/19	11/19	1/6	SB	serum	QDB kit	NR	PCR	*IL1B*
Combaret et al.^15^	Europe, USA	10/24	16/27	41/83	6/60	SB/FISH	serum	QDB kit	5	qPCR	*NAGK*
Kojima et al.^16^	Japan	0/20	2/7	14/6	0/1	SB/FISH	plasma	QDB kit	2–5	qPCR	*NAGK*
Yagyu et al.^17^	Japan, USA	6/38	12/14	38/33	1/6	SB/FISH	serum	QDB kit	5	qPCR	*NAGK*
Ma et al.^18^	South Korea	0/31	1/13	9/49	0/2	FISH	serum	QDB kit	1.6	PCR	*NAGK*

*FISH* fluorescence in situ hybridisation, *NR* not reported, *SB* Southern blot, *QDB kit* QIAamp DNA Blood kit.

### Diagnostic accuracy of cfDNA-based MNA analysis

An initial analysis was conducted across all tumour stages (Supplementary Table 1). Since very few patients with localised (stage 1 and 2) or stage 4S disease were recruited to the seven studies, and MNA is uncommon, these patient sub-groups could not be reliably meta-analysed and were therefore excluded. Sensitivity, specificity and likelihood ratios for the remaining 529 patients with advanced-stage (stage 3 and 4) disease are reported for each study in Table 2.

**Table 2:** DOR, sensitivity, specificity and likelihood ratios with calculated 95% confidence intervals for each study in patients with advanced-stage (stage 3 and 4) NB.

Study	DOR (95% CI)	Sens. (95% CI)	Spec. (95% CI)	PLR (95% CI)	NLR (95% CI)
Combaret et al.^13^	1160.0 (69.7–19320.1)	0.97 (0.83–0.99)	0.98 (0.87–1.00)	39.6 (5.7–275.0)	0.03 (0.01–0.24)
Gotoh et al.^14^	1581.0 (29.8–83804.6)	1.00 (0.80–1.00)	1.00 (0.87–1.00)	50.4 (3.2–785.2)	0.03 (0.00–0.49)
Combaret et al.^12^	117.0 (14.9–918.0)	0.87 (0.62–0.96)	0.95 (0.83–0.99)	16.5 (4.2–64.4)	0.14 (0.04–0.51)
Combaret et al.^15^	999.8 (57.4–17411.0)	0.82 (0.71–0.90)	1.00 (0.97–1.00)	181.8 (11.4–2896.3)	0.18 (0.11–0.31)
Kojima et al.^16^	891.0 (16.6–7940.6)	1.00 (0.81–1.00)	1.00 (0.77–1.00)	27.2 (1.8–413.8)	0.03 (0.00–0.47)
Yagyu et al.^17^	107.6 (25.3–457.4)	0.88 (0.76–0.94)	0.94 (0.83–0.98)	13.8 (4.6–41.4)	0.13 (0.06–0.27)
Ma et al.^18^	270.0 (22.2–3291.3)	0.90 (0.60–0.98)	0.97 (0.89–0.99)	27.9 (7.0–110.8)	0.10 (0.02–0.66)

*DOR* diagnostic odds ratio, *NLR* negative likelihood ratio, *PLR* positive likelihood ratio, *Sens* sensitivity, *Spec* specificity.

**Fig. 2:**
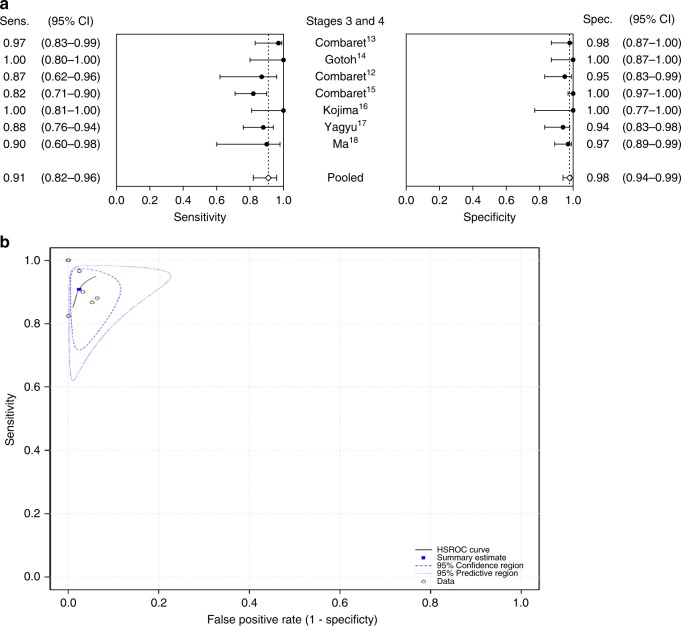
Estimated pooled sensitivity, specificity and hierarchical summary receiver operator characteristic (HSROC) curve in advanced-stage patients. **a** Forest plots of sensitivity and specificity of cfDNA-based MNA analysis at diagnosis in NB patients with advanced-stage disease. **b** HSROC curve analysis for patients with advanced-stage (stage 3 and 4) disease.

**Fig. 3:**
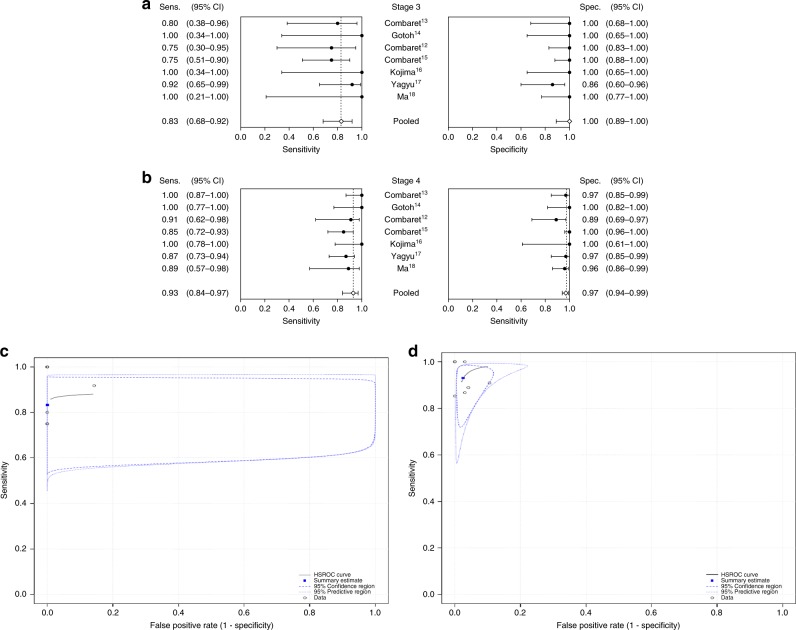
Estimated pooled sensitivity, specificity and hierarchical summary receiver operator characteristic (HSROC) curve in stage 3 and stage 4 patients. **a**, **b** Forest plots of sensitivity and specificity of cfDNA-based MNA analysis at diagnosis in NB patients with **a** stage 3 and **b** stage 4 disease. **c**, **d** HSROC curve analysis for patients with **c** stage 3 and **d** stage 4 disease.

Further, we calculated estimated pooled data and performed sub-group analysis (Figs. 2 and 3; Tables 3 and 4). Specifically, using a bivariate random-effects model, the estimated pooled sensitivity of cfDNA was 0.908 (95% CI, 0.818–0.956) and the estimated pooled specificity was 0.976 (0.940–0.991) (Fig. 2a; Table 4). Estimates of the pooled positive and negative likelihood ratios (PLR, NLR) were 38.6 (1.8–75.5) and 0.094 (0.027–0.161), respectively. The pooled diagnostic odds ratio (DOR) was 410.0 (−103.6 to 923.7) (Table 4) and the pooled hierarchical summary receiver operator characteristic (HSROC) curve was calculated (Fig. 2b).

**Table 3:** DOR, sensitivity, specificity and likelihood ratios with calculated 95% confidence intervals for each study in patients sub-grouped by INSS stage.

Study	DOR (95% CI)	Sens (95% CI)	Spec (95% CI)	PLR (95% CI)	NLR (95% CI)
INSS stage 3					
Combaret et al.^13^	51.0 (1.70–1525.8)	0.80 (0.38–0.96)	1.00 (0.68–1.00)	13.5 (0.9–207.6)	0.265 (0.066–1.068)
Gotoh et al.^14^	75.0 (1.16–4868.6)	1.00 (0.34–1.00)	1.00 (0.65–1.00)	13.3 (0.9–204.7)	0.178 (0.014–2.247)
Combaret et al.^12^	91.0 (3.05–2718.1)	0.75 (0.30–0.95)	1.00 (0.83–1.00)	28.0 (1.7–458.8)	0.308 (0.081–1.176)
Combaret et al.^15^	152.8 (7.60–3060.2)	0.75 (0.51–0.90)	1.00 (0.88–1.00)	41.2 (2.6–651.7)	0.270 (0.122–0.596)
Kojima et al.^16^	75.0 (1.16–4868.6)	1.00 (0.34–1.00)	1.00 (0.65–1.00)	13.3 (0.9–204.7)	0.178 (0.014–2.247)
Yagyu et al.^17^	66.0 (5.20–833.6)	0.92 (0.65–0.99)	0.86 (0.60–0.96)	6.4 (1.8–23.4)	0.097 (0.015–0.643)
Ma et al.^18^	81.0 (1.14–5778.7)	1.00 (0.21–1.00)	1.00 (0.77–1.00)	21.0 (1.2–358.4)	0.259 (0.023–2.865)
INSS stage 4					
Combaret et al.^13^	1105.0 (43.2–28280.7)	1.00 (0.87–1.00)	0.97 (0.85–0.99)	22.2 (4.6–106.4)	0.020 (0.001–0.313)
Gotoh et al.^14^	999.0 (18.63–53582.1)	1.00 (0.77–1.00)	1.00 (0.82–1.00)	36.6 (2.4–565.8)	0.037 (0.002–0.558)
Combaret et al.^12^	85.0 (6.81–1061.0)	0.91 (0.62–0.98)	0.89 (0.69–0.97)	8.6 (2.3–32.5)	0.102 (0.016–0.663)
Combaret et al.^15^	912.08 (50.0–16628.0)	0.85 (0.72–0.93)	1.00 (0.96–1.00)	142.0 (8.9–2258.4)	0.156 (0.077–0.316)
Kojima et al.^16^	377.0 (6.7–21160.0)	1.00 (0.78–1.00)	1.00 (0.61–1.00)	13.5 (0.9–195.9)	0.036 (0.002–0.552)
Yagyu et al.^17^	211.2 (23.4–1908.8)	0.87 (0.73–0.94)	0.97 (0.85–0.99)	28.7 (4.1–198.2)	0.136 (0.060–0.308)
Ma et al.^18^	188.0 (15.2–2324.4)	0.89 (0.57–0.98)	0.96 (0.86–0.99)	21.8 (5.5–86.3)	0.116 (0.018–0.736)

*DOR* diagnostic odds ratio, *NLR* negative likelihood ratio, *PLR* positive likelihood ratio, *Sens* sensitivity, *Spec* specificity.

**Table 4:** Summary of the diagnostic accuracy of MNA assessment in cfDNA of patients with INSS stage 3 and/or 4 NB with calculated 95% confidence intervals.

INSS stage	Sens (95% CI)	Spec (95% CI)	PLR (95% CI)	NLR (95% CI)	DOR (95% CI)
3 and 4	0.908 (0.818–0.956)	0.976 (0.940–0.991)	38.6 (1.8–75.5)	0.094 (0.027–0.161)	410.0 (−103.6–923.7)
3	0.832 (0.677–0.921)	0.999 (0.109–1.000)	1321.2 (−11172.2–13814.6)	0.168 (0.048–0.288)	7855.2 (−66267.0–81977.4)
4	0.930 (0.838–0.972)	0.974 (0.937–0.990)	36.4 (3.6–69.3)	0.072 (0.009–0.134)	508.7 (−85.8–1103.2)

*DOR* diagnostic odds ratio, *NLR* negative likelihood ratio, *PLR* positive likelihood ratio, *Sens* sensitivity, *Spec* specificity.

To determine whether disease stage could significantly influence the accuracy of cfDNA-based MNA analysis, stage 3 and stage 4 patients were subjected to sub-group analyses. Per-study sensitivity, specificity and likelihood ratios for each stage are shown in Table 3. The estimated pooled sensitivity of cfDNA for patients with stage 3 and 4 disease was 0.832 (0.677–0.921) and 0.930 (0.838–0.972), respectively, and the pooled specificity was 0.999 (0.109–1.000) and 0.974 (0.937–0.990), respectively (Fig. 3a, b; Table 4). The pooled PLR for stage 3 and 4 patients was 1321.2 (−11172.2 to 13814.6) and 36.4 (3.6–69.3), respectively, and the pooled NLR was 0.168 (0.048–0.288) and 0.072 (0.009–0.134), respectively (Table 4). The pooled DOR was 7855.2 (−66267.0 to 81977.4) and 508.7 (−85.8 to 1103.2), respectively, and the pooled HSROC curves were generated (Fig. 3c, d).

### Assessment of threshold effect and publication bias

**Fig. 4:**
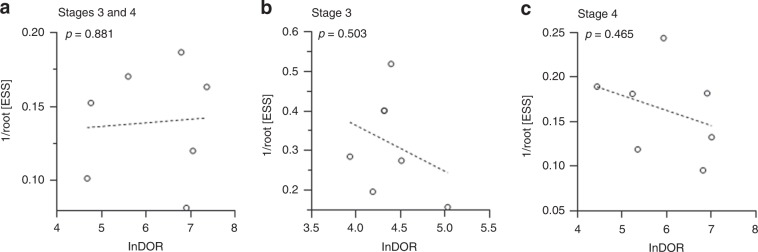
Assessment of publication bias. Deeks’ funnel plots of DOR for cfDNA-based MNA analysis in **a** stage 3 and 4, **b** stage 3 and **c** stage 4 NB patients. Each point represents the natural logarithm of the DOR of a study plotted against the square root of its effective sample size (ESS).

A Spearman’s correlation coefficient of −0.126 (*p* = 0.788) between sensitivity and 1-specificity indicated the absence of a threshold effect among the included studies. Furthermore, the ROC plane did not show a curvilinear pattern characteristic of a threshold effect (data not shown). Further investigation of DOR revealed low heterogeneity due to non-threshold effect (data not shown). The potential for publication bias was visually assessed by Deeks’ funnel plot and statistically calculated by Deeks’ asymmetry test.^29^ No significant bias was found among the studies for stage 3 and 4 combined *(p* = 0.881), stage 3 alone (*p* = 0.503) and stage 4 alone (*p* = 0.465) (Fig. 4).

### Assessment of study quality

**Fig. 5:**
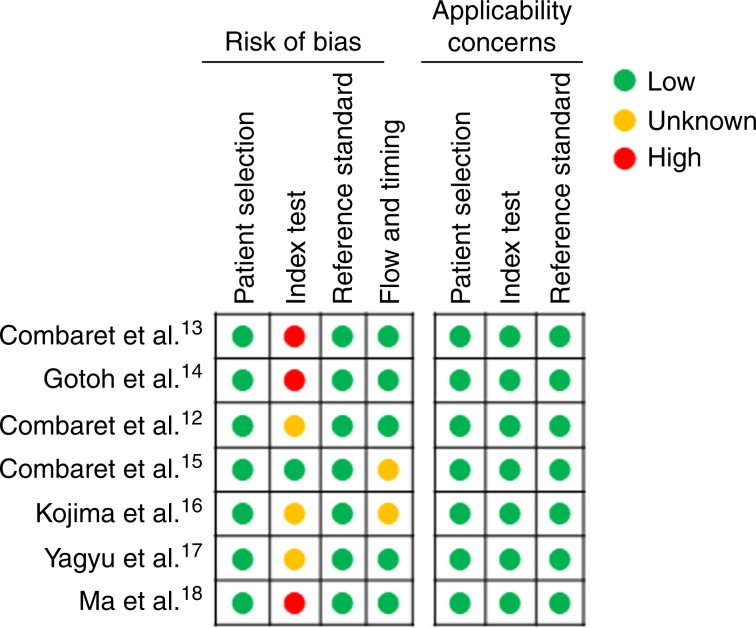
Quality assessment of studies by QUADAS-2.

The overall quality of the studies included in this meta-analysis was evaluated with QUADAS-2^21^ (Fig. 5). This tool was designed to evaluate individual studies on the basis of patient selection, index test, reference standard, and flow and timing. Study quality was generally high with a low risk of bias and low concerns of applicability. However, none of the studies determined the MNA cut-off prior to analysis, and in three studies it was not specified whether the cfDNA analyses were conducted in a blind manner or with prior knowledge of tissue MNA status (reference standard).

## Discussion

MNA status is a critical factor that informs the prognostic and therapeutic course of patients with NB.^2^ To overcome several limitations of MNA analysis in biopsy tissue at diagnosis, studies over the past two decades have investigated the utility of cfDNA in plasma or serum as a tumour surrogate.^30^ The aim of this meta-analysis was to determine the diagnostic accuracy of MNA analysis in cfDNA of patients with NB using FISH or Southern blot as the reference standard and a PCR method as the index test. The comprehensive search strategy identified 12 studies, of which 7 were suitable for inclusion, assessing a total of 844 patients of all INSS stages. Reflecting the very low incidence of MNA in patients with stage 1, 2 and 4S disease,^31–33^ the seven included studies individually recruited few or no MNA-positive patients from these stage groups. Therefore, to avoid introducing significant bias to the analysis, this study did not include stage 1, 2 or 4S patients in the pooled or sub-group analyses, leaving 529 patients with advanced-stage (stage 3 and 4) disease.

For patients with advanced-stage disease, pooled analysis showed that MNA status was determined with high sensitivity and almost perfect specificity (0.908 and 0.976, respectively). Consequently, the diagnostic accuracy was very high, with a DOR of 410.042. Given that the tumour-derived fraction of cfDNA increases with tumour burden in many solid cancers including NB,^34,35^ it was considered necessary to perform a sub-group analysis on patients with stage 3 and stage 4 disease. While the specificity for both patient sub-groups were 0.999 and 0.974 for stage 3 and 4, respectively, sensitivity was lower for patients with stage 3 disease relative to stage 4 (0.832 vs. 0.930, respectively). This resulted in a higher global performance for metastatic disease, as expected, given the high tumour burden in these patients. It is noteworthy that while the rate of false positives in this meta-analysis was very low among stage 3 and 4 patients (2/137 and 6/392, respectively), these occurrences may be attributable to intratumoural heterogeneity with respect to MNA, leading to a negative result by FISH analysis of tissue and a positive result by PCR analysis of cfDNA.

None of the included studies determined the cut-off *MYCN*/reference gene ratio to define MNA prior to analysis, and the implemented cut-off ratios were either wide-ranging or unreported. However, a threshold effect was ruled out by Spearman’s correlation coefficient and visual ROC plane analysis. Other aspects of the study design were generally acceptable according to the QUADAS-2 framework.^21^ A distinct strength of this meta-analysis is the consistency in index test methodology between studies; all studies isolated cfDNA using the same commercial kit, employed a PCR technique and normalised *MYCN* to a single reference gene. A potential source of heterogeneity was in the use of plasma vs. serum as a source of cfDNA; whereas cfDNA in plasma is stable for several hours post-venepuncture, a delay in processing of serum as well as contamination by white blood cells can result in the release of genomic DNA into the sample, thus potentially masking detection of *MYCN* gene amplification by high levels of DNA from normal cells.^36^

The high diagnostic accuracy of cfDNA in advanced-stage patients, as demonstrated in this study, has promising implications for several clinical scenarios. In patients with surgically inaccessible tumours, or in patients who are critically unwell, a biopsy may not be possible,^12^ whereas blood collection is less invasive and repeatable if insufficient material is obtained at first attempt.^37^ Moreover, the rapidity of blood collection, automated cfDNA extraction and simple analysis enables fast determination of MNA status in patients who require immediate assignment to appropriate treatment. Analysis of cfDNA is also advantageous over tissue analysis in tumours exhibiting heterogeneous patterns of MNA;^6,7^ cfDNA may also have the potential to reveal MNA in patients with heterogeneity between their primary tumour and metastases^38^ and provide a critical opportunity for additional therapeutic intervention. As with all technologies, there are limitations to this approach, as it requires that sufficient molecules are present in the plasma or serum at the time of collection, which may not be the case in patients with intratumoural heterogeneity and small, early-stage tumours.

While stage 4S disease was excluded from this meta-analysis, MNA is relativity uncommon in these patients and its prognostic significance is disputed.^33,39–41^ In contrast, MNA is firmly established as a poor prognostic indicator in patients with stage 1 and 2 disease, albeit occurring at a frequency of only 3–4%.^31,32^ Of the seven included studies, only four patients with MNA-positive stage 1 and 2 disease were reported. Combaret et al.^15^ reported a very low sensitivity of cfDNA analysis in stage 1 and 2 patients, with only one patient showing evidence of MNA in cfDNA among 10 patients with MNA-positive tumours.^15^ This observation is not unexpected, given evidence from other early-stage solid cancers to indicate that low tumour burden limits the detectability of tumour-specific alterations in cfDNA,^42,43^ particularly copy number alterations due to the dilution effect of cfDNA derived from apoptosis of healthy blood cells. It is also noteworthy that genomic DNA contamination arising from lysed white blood cells with the delayed processing of serum is likely to disproportionately influence the sensitivity of MNA analysis in early-stage NB patients. Hence, future studies recruiting patients with stage 1 and 2 disease should consider plasma as the preferred specimen type.

Molecular diagnostic laboratories are increasingly becoming equipped with next-generation sequencing platforms, and in the future, it may be possible to employ sequencing-based methods for analysis of MNA along with other prognostic or actionable genomic alterations in cfDNA. To this end, it has recently been shown that MNA among other alterations can be detected in the cfDNA of NB patients using shallow whole-genome/exome sequencing^44,45^ and microarray methods.^46^ However, these studies must be replicated with larger patient cohorts in a diagnostic setting before a meta-analysis can be undertaken.

## Conclusion

In conclusion, this is the first systematic review and meta-analysis of the diagnostic performance of cfDNA for the determination of MNA status in patients with advanced-stage NB. The studies assessed used simple and widely available tests (PCR or qPCR), highlighting the potential of implementing a straightforward and inexpensive blood-based diagnostic test for use in patients who are too unwell for surgery or where biopsy is not possible. Standardised methodology for cfDNA analysis should be developed and incorporated into future large-scale prospective trials for clinical validation and to determine the effects of therapy on plasma/serum MNA status.

## Supplementary information

Supplementary information 1

Supplementary table 1

## Data Availability

All data generated and analysed during this study are included in this published article and its supplementary information files.
