# Supplementary material for: Diagnostic accuracy of circulating-free DNA for the determination of MYCN amplification status in advanced-stage neuroblastoma: a systematic review and meta-analysis
Source: Br J Cancer. 2020 Feb 4;122(7):1077–84. doi: 10.1038/s41416-020-0740-y (PMC7109036; doi:10.1038/s41416-020-0740-y)
Supplement: Supplementary file 1 — Supplementary information 1 [file 41416_2020_740_MOESM1_ESM.docx]

**Supplementary Information 1**. Search strings used for each database.

**CENTRAL**

#1 [Title/Abstract/Keyword] Neuroblastoma

*AND*

#2 [Title/Abstract/Keyword] MYCN

*AND*

#3 [Title/Abstract/Keyword] “circulating-free DNA” OR ccfDNA OR cfDNA OR ctDNA OR “cell-free DNA” OR “cell free DNA” OR “circulating DNA” OR “circulating free DNA” OR “circulating tumo?r DNA” OR “free DNA” OR “free tumo?r DNA” OR plasma OR serum

Article *n* = 8

**EMBASE**

#1 [Abstract] Neuroblastoma

*AND*

#2 [Abstract] MYCN

*AND*

#3 [Abstract] circulating-free DNA OR ccfDNA OR cfDNA OR ctDNA OR cell-free DNA OR cell free DNA OR circulating DNA OR circulating free DNA OR circulating tumo?r DNA OR free DNA OR free tumo?r DNA OR plasma OR serum

Article *n* = 153

**PubMed/MEDLINE**

#1 [Title/Abstract] Neuroblastoma

*AND*

#2 [Title/Abstract] MYCN

*AND*

#3 [Title/Abstract] “circulating-free DNA” OR ccfDNA OR cfDNA OR ctDNA OR “cell-free DNA” OR “cell free DNA” OR “circulating DNA” OR “circulating free DNA” OR “circulating tumor DNA” OR “circulating tumour DNA” OR “free DNA” OR “free tumor DNA” OR “free tumour DNA” OR plasma OR serum

Article *n* = 101

**Web of Science Conference Proceedings Citation Index – Science (CPCI-S)**

#1 [Topic] Neuroblastoma

*AND*

#2 [Topic] MYCN

*AND*

#3 [Topic] “circulating-free DNA” OR ccfDNA OR cfDNA OR ctDNA OR “cell-free DNA” OR “cell free DNA” OR “circulating DNA” OR “circulating free DNA” OR “circulating tumo$r DNA” OR “free DNA” OR “free tumo$r DNA” OR plasma OR serum

Article *n* = 12
