# Supplementary material for: Diagnostic accuracy of circulating-free DNA for the determination of MYCN amplification status in advanced-stage neuroblastoma: a systematic review and meta-analysis
Source: Br J Cancer. 2020 Feb 4;122(7):1077–84. doi: 10.1038/s41416-020-0740-y (PMC7109036; doi:10.1038/s41416-020-0740-y)
Supplement: Supplementary file 2 — Supplementary table 1 [file 41416_2020_740_MOESM2_ESM.docx]

| TP FN  FP TN | **INSS stage** | | | | | | | | | | | | | |
| --- | --- | --- | --- | --- | --- | --- | --- | --- | --- | --- | --- | --- | --- | --- |
|  | **1** | | **2** | | **1+2** | | **3** | | **4** | | **4S** | | **Overall** | |
| **Combaret**  **et al. 2002** | 0 | 0 | 1 | 0 | 1 | 0 | 4 | 1 | 25 | 0 | 1 | 0 | 31 | 1 |
|  | 0 | 14 | 0 | 10 | 0 | 24 | 0 | 8 | 1 | 32 | 0 | 5 | 1 | 69 |
| **Gotoh**  **et al. 2005** | 1 | 0 | 1 | 0 | 2 | 0 | 2 | 0 | 13 | 0 | 0 | 0 | 17 | 0 |
|  | 0 | 22 | 0 | 18 | 0 | 40 | 0 | 7 | 0 | 18 | 0 | 5 | 0 | 70 |
| **Combaret**  **et al. 2005** | 0 | 0 | 0 | 0 | 0 | 0 | 3 | 1 | 10 | 1 | 1 | 0 | 14 | 2 |
|  | 0 | 17 | 0 | 8 | 0 | 25 | 0 | 19 | 2 | 17 | 0 | 6 | 2 | 67 |
| **Combaret**  **et al. 2009** | 1 | 3 | 0 | 6 | 1 | 9 | 12 | 4 | 35 | 6 | 5 | 1 | 53 | 20 |
|  | 0 | 15 | 0 | 9 | 0 | 24 | 0 | 27 | 0 | 83 | 0 | 60 | 0 | 194 |
| **Kojima**  **et al. 2013** | 0 | 0 | 0 | 0 | 0 | 0 | 2 | 0 | 14 | 0 | 0 | 0 | 16 | 0 |
|  | 0 | 16 | 0 | 4 | 0 | 20 | 0 | 7 | 0 | 6 | 0 | 1 | 0 | 34 |
| **Yagyu**  **et al. 2016** | - | - | - | - | 4 | 2 | 11 | 1 | 33 | 5 | 1 | 0 | 49 | 8 |
|  | - | - | - | - | 2 | 36 | 2 | 12 | 1 | 32 | 0 | 6 | 5 | 86 |
| **Ma**  **et al. 2016** | 0 | 0 | 0 | 0 | 0 | 0 | 1 | 0 | 8 | 1 | 0 | 0 | 9 | 1 |
|  | 0 | 10 | 0 | 21 | 0 | 31 | 0 | 13 | 2 | 47 | 0 | 2 | 2 | 93 |
| **Pooled** | - | - | - | - | 8 | 11 | 35 | 7 | 138 | 13 | 8 | 1 | 189 | 32 |
|  | - | - | - | - | 2 | 200 | 2 | 93 | 6 | 235 | 0 | 85 | 10 | 613 |

**Supplementary Table 1**. 2x2 contingency tables for patients per study, grouped by INSS tumour stage. -, stage 1 and 2 are reported in combination. TP, true positive; FN, false negative; FP, false positive; TN, true negative.
